# Reducing pediatric caries and obesity risk in South Asian immigrants: randomized controlled trial of common health/risk factor approach

**DOI:** 10.1186/s12889-018-5317-9

**Published:** 2018-05-31

**Authors:** Alison Karasz, Karen Bonuck

**Affiliations:** Department of Family and Social Medicine, Albert Einstein College of Medicine, Bronx, New York 10461 USA

**Keywords:** Obesity, Oral health, Caries, Children, Common risk factor approach, South Asian

## Abstract

**Background:**

This paper describes the design and methods of a multi-phase study to reduce early childhood caries and obesity in vulnerable South Asian (SA) immigrants in the United States. Early childhood caries and obesity are the most common diseases of early childhood. Risk factors for both diseases are rooted in early childhood feeding practices such as bottle feeding and intake of sweets and sweetened beverages. The Common Health/Risk Factor Approach to addressing oral health is widely promoted by the WHO and other policy makers. This approach recognizes links between oral health and other diseases of modernity. Our CHALO! (“Child Health Action to Lower Obesity and Oral health risk”--from a Hindi word meaning "Let's go!") study targets SA families at high risk for early childhood caries and obesity. CHALO! addresses common risk factors associated with these two common diseases of childhood.

**Methods:**

This two part project includes a randomized controlled trial, and a Knowledge Translation campaign. A randomized controlled trial will enroll *n* =  360 families from pediatric practices serving South Asians in the New York metro area. The intervention group will receive home visits by SA community health workers at 6, 8, 10, 12, 14, and 16 months of age. Controls will receive culturally tailored educational material. Primary outcomes-- cariogenic and obesogenic feeding practices at 6, 12, and 18 months-- will be assessed with the MySmileBuddy iPad based tool. Secondary outcomes include: oral hygiene practices, anthropometrics, and caries incidence at 18 months. A public education campaign will focus on both families and health care providers.

**Discussion:**

There are few Common Health/Risk Factor Approach published studies on obesity and oral health risk in children, despite health morbidity and costs associated with both conditions. CHALO! comprises a multi-level interventions designed to promote culturally competent, sustainable change.

**Trial registration:**

ClinicalTrials.gov NCT03077425.

**Electronic supplementary material:**

The online version of this article (10.1186/s12889-018-5317-9) contains supplementary material, which is available to authorized users.

## Background

Immigrants from the developing world are impacted by the global *nutrition transition,* from low calorie, highly nutritious foods in resource-poor environments, to the high calorie, low nutrition diets of the west [1]. Nutrition-related diseases occur with the adoption of western diets. Obesity and oral caries, the most common diseases of early childhood in western societies, are prevalent in low income immigrant communities [2–4]. The Child Health Action to Lower risk of Obesity and Oral caries--**CHALO!** (“let’s go!” in Hindi)-- is a multi-level intervention culturally tailored for at-risk immigrant South Asians (SAs) from India, Pakistan, and Bangladesh. These SAs are among the fastest growing immigrant groups in the US [5, 6].

SAs are particularly vulnerable to nutrition-related disease. A modernizing diet in urban South Asian has created a public health ‘tsunami’ of obesity and metabolic disorders in that region [7–9]. SA immigrants in the UK and Canada bear a high burden of obesity-related health disparities [10–12]. SA immigrant children demonstrate growth patterns associated with metabolic risk: low birthweight followed by steep weight gain in infancy and childhood [13]. A tendency towards central adiposity places adults and children at high risk for metabolic syndrome even controlling for body mass index (BMI) [9, 14]. Children are also at high risk for early childhood caries (ECC) [15–17], even adjusting for socio-economic status s [18].

### The common risk factor approach

The Common Risk/Health Factor Approach (CR/HFA) to oral health disparities focuses on risk factors and underlying social determinants shared with other diseases of modernity [19]. Based on the CR/HFA, CHALO! employs a multi-level social-ecological framework for conceptualizing and addressing oral health and obesity risk (See Table 1):

**Table 1:** Outline CHALO Multi Level Intervention

Activity	Target	Level
Home Visit with Mothers	Knowledge, support, wellbeing, confidence, skills, feeding behaviors	Individual
Home Visits with Families	Family/household support for Mothers’ Goals	Interpersonal
Dental Referral & Navigation	Link families to providers; facilitate appointments	Organizational
Knowledge Translation --Community	Raise awareness & educate via: 1-to-1 outreach, local meetings, video/drama	Community
Professional Dissemination	Educate local providers on AAP & AAPD policy; raise cultural awareness; promote professionals-researcher-policymaker dialogue	Community & Policy

#### Individual level influences

Maternal feeding practices in early life increase the risk of caries (i.e., cariogenic) and obesity (i.e., obesogenic). They include: prolonged use of bottles (and ‘sippy cups’); adding solids and sweets to bottles; nighttime feeding; salty and starchy snacks; sweets, and sweetened beverages. Bottle use beyond 12–15 months increases risk for both conditions [20–26], as do bottles at night or naptime [22] or bottles that contain solids [27] and sweeteners [28]. Bottle feeding *facilitates overriding of a child’s internal satiety cues,* [29] thereby reducing ability to self-regulate intake [30]. This may further promote both weight gain [31, 32] and early childhood caries (ECC) [24].

Such feeding practices are common in SA communities [33, 34]. Up to 75% of low-income SA 15-month olds still use a bottle, and 30% regularly drink sweetened milk [35–37]. *Non-responsive feeding* is also common in SA families. Non-responsive feeding’s defining feature is caregiver lack of responsiveness to a child’s feeding cues. It includes both adult controlling (forcing or restricting) and child controlling (indulgent) feeding [38]. Forcing/controlling feeding leads to rapid weight gain in the first year of life, which predispose to overweight in later years [39, 40]. Indulgent styles are associated with obesity in toddlers and older children [41]. Among SAs in the New York area, ‘force feeding’ is routine. Once children are old enough to ask for food, force-feeding yields to indulgent feeding. SA mothers fear distressing their children by refusing food [42].

In addition to common risk behaviors, there are unique risk behaviors associated with obesity and caries that are common among SA immigrants. SA children are more sedentary than their white counterparts, raising their risk of obesity [43–46]. Oral health prevention—e.g., brushing, oral hygiene, and preventive dental care—are undervalued in SA communities. Many families seek dental care only when there is pain or visible decay; baby teeth may be viewed as dispensable [42, 47, 48].

#### Interpersonal level influences

SA married women’s low status and limited access to family decision-making may impede adherence to medical advice [42]. Senior family members may insist traditional feeding patterns including frequent sweets and delayed weaning [37, 42]. Maternal depression—due to factors such as poverty, isolation, and marital abuse—is common in SA families [49–51]. Maternal depression is associated with non-responsive feeding [52, 53] and child obesity [41]. Poverty and its sequelae, including substandard housing, further limit mothers’ ability to change feeding patterns. Such may cause disruption and crying, which may be difficult to tolerate in crowded living quarters [42].

#### Institutional/organizational level influences

Like other low-income immigrants, SA children lack access to dental care [4, 54, 55]. Pediatricians rarely refer to dentists [4, 54–57]. Guidelines recommend a first dental visit by 12 months of age and two visits per year for low-income and immigrant children [58, 59]. Yet only 1.5% of one-year olds visit a dentist each year [60] and rates are lowest in poor families who could most benefit [61]. Another barrier to care for low income children is the fact that many dentists on the US don’t accept Medicaid or Children’s Health Insurance Program coverage [62].

#### Community level influences

These include cultural beliefs and practices, along with low awareness of risk factors. Though concerns over child feeding are widely shared among SA mothers, there is relatively little understanding of how non-responsive feeding leads to child food refusal, creating a vicious cycle of increased maternal anxiety and force feeding. One SA mother on www.indusladies.com, a website for South Asian moms, asks: "Why do American kids happily feed themselves from the age of one year old and are constantly asking for food? Why do Indian kids hate food and need to be FORCE FED until the age of 4,5,6, or even 7?" Pediatricians in the SA community often report frustration. “*Feeding problems take up 75% of my time*.” (Mehotra N, SAPPHIRE network pediatrician. In.; 2015, personal communication). Physicians lack tools and support needed to address this problem. *“The most commonly prescribed medication in my practice is an appetite stimulant. Even though the child doesn’t need it, I have to prescribe to get peace in the family.”* (Mehotra N. In.; 2015, personal communication).

#### Policy level influences

The Knowledge Translation campaign will work with private practitioners, residents, and teaching faculty in dental and pediatric departments. Through dialogue and presentations in clinical and educational settings, the goal is to develop new best practices and guidelines regarding the improved care of South Asian immigrant children.

The randomized controlled trial (RCT), CHALO! will answer the following research questions: a) Does a culturally-tailored CR/HFA home-visiting intervention, from child age 6 through 16 months, reduce cariogenic and obesogenic behaviors in SA immigrant families (primary hypothesis)? b) Is this CR/HFA intervention associated with reduced incidence of oral caries and obesity at 18 months of age (secondary hypothesis)?

## The CHALO project RCT

### Methods

To address individual and interpersonal (i.e., household) risk factors, SA community health workers (CHWs) will conduct a home-visiting intervention. Home Visiting is a decades-old strategy to improve maternal-child health outcomes [63–65] by empowering families. We will recruit low-income SA mother-child dyads from pediatric practices in New York City and New Jersey (U.S.). Inclusion criteria include: a) *Age*: Child is < 6 months old at time of recruitment; b) *Insurance*: Child is enrolled in either Medicaid or the Children’s Health Insurance Program; c) *Nativity*: Mother was born in India, Pakistan, or Bangladesh); d) *Language*: Mother speaks standard Bengali, English or Hindi/Urdu; e) *Agency*: Mother is index child’s primary caretaker. Exclusion criteria include: a) Inability to provide informed consent; b) Plans to travel for > 1 month during follow-up, and; c) child health condition barring participation (per pediatrician review).

#### Enhanced usual care (control)

An EUC counseling intervention will be delivered immediately following the T0 Baseline Interview, just prior to randomization. EUC Components include: 1) a pamphlet- containing basic ECC and Obesity prevention messages, and 2) Dental Referrals. We developed a Screened Dental Provider list of dentists who: a) are willing to see 12 month old children; and b) accept most insurance plans in the study practices.

#### Intervention

CHWs will conduct *n* = 6 home visits when the child is 6, 8, 10, 12, 14, and 16 months old. Of these, 4 visits include only the mother-child dyad. Visits at 8 and 14 months will include a senior family decision maker (e.g., husband or mother-in-law). CHWs will conduct 6 follow up telephone calls in alternate months. Calls will be used to ask about the family’s goals for change, gauge progress, and assist with trouble shooting.

**Fig. 1:**
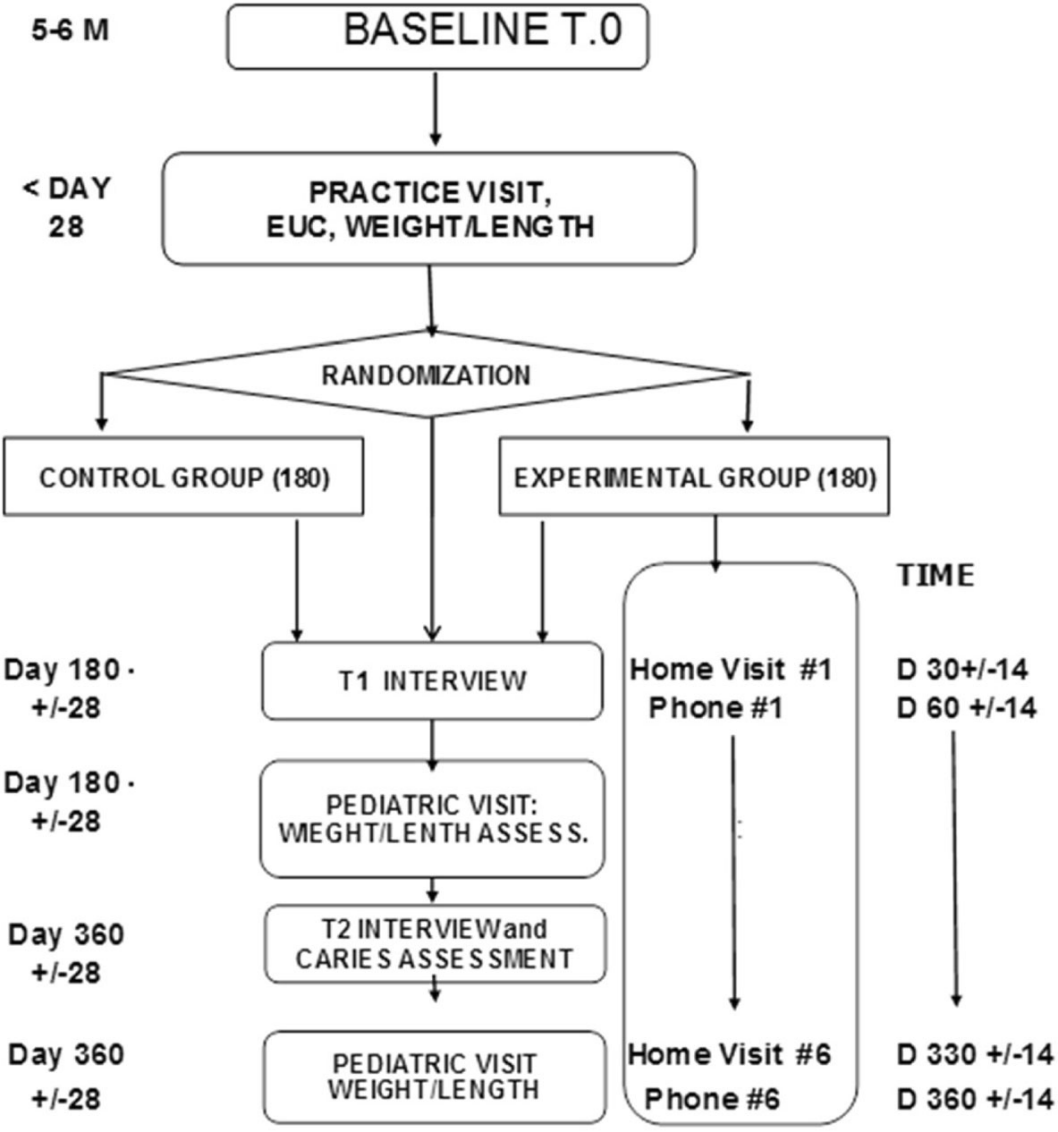
Study flow

*Mother-only visits* will focus on building rapport and intimacy, identifying family/household concerns, reviewing risk/health behaviors, reviewing, setting, and troubleshooting goals, building skills through active interaction with the child, and providing education. Messages will be provided orally and via culturally tailored, attractive educational materials. To bolster mothers’ skills, CHW-guided interactions with the dyads will include: recognizing infant satiety and hunger cues via responsive bottle feeding, preparing age-appropriate self-feeding foods, encouraging tummy time and active play, transitioning from naptime milk to water bottles, and oral hygiene practices such as tooth brushing. At each visit, the CHW encourages the mother to set a concrete, manageable behavior change goal. Patient navigation, as needed, will encourage mothers to make and keep two dental visits for their child, over the 12 month follow-up. *Family visits* will, similarly, assess family views on barriers to change, and enlist support for mothers’ child feeding goals (Fig. 1).

### Outcomes measurement:

The RCT’s **primary aim** is to reduce risk behaviors for caries and obesity. We hypothesize that relative to Controls, the Intervention group will report: less bottle use (any, with added sweeteners/solids, at nap and bedtimes), improved food choices (*more* fruits and vegetables, *less* juice and sweetened beverages, *fewer* sweet and salty snacks); *more* physical activity and *less* screen time; and *more* tooth brushing and dental visits. See Table 2 for Schedule of Research Assessments.

**Table 2:** Schedule of Research Assessments: 6, 12, and 18 Months

Interview	Data Obtained	Setting
Baseline @ 6 mos. (T0)	Questionnaires	Home
	MySmileBuddy (MSB)	
	Weight/length	
12 mo. Interview (T1)	Questionnaires	Home
	MySmileBuddy (MSB)	
	Weight/length	
18 mo. Interview (T2)	Questionnaires	Home
	MySmileBuddy (MSB)	
	Caries Exam	
	Weight/length	

#### Common risk factor feeding behaviors

We will use MySmileBuddy (MSB), an iPad based ECC risk assessment and educational tool [66]. MSB was validated in previous research [67]. The MSB guides parents through a modified 24-h dietary recall for their child. We have adapted the MSB to reflect culturally appropriate SA foods and beverages, as well as the types of bottles and cups used for beverages. Participants are cued by queries (e.g., first thing to eat or drink upon waking) and attractive color photographs of food and beverage groupings. Prior to administration, the research assistant will ask whether the prior 24 h represented a ‘usual day.’ If not, participants will conduct a recall for one day earlier. We will conduct a second 24-h recall for a subset of participants.

*Unique risk factors for obesity* (sedentary behavior/screen time) will be assessed through previously validated questionnaire items used in other large studies. *Unique ECC-risk factors* include dental visits and oral hygiene practices. We will assess frequency of tooth brushing, use of toothpaste, and use of tap water using items from a measure developed by the CDC. Dental health utilization will be assessed through questions at T1 and T2.

The RCT’s **secondary aim** is to reduce the velocity of weight gain in months 12–18, and the incidence of oral caries at 18 months. Weight and length data will be collected by research assistants at the T0, T1, and T2 outcomes assessments. Study pediatricians will train research staff in anthropometrics protocol. For length, two supine measures will be taken using infantometers (Seca 417, Brooklyn NY), to the nearest 0.1 cm. For weight, children will be measured with diaper and light clothing. ECC will be assessed by study staff using Intraoral cameras at the 18 month follow-up. Intraoral cameras are valid [68–72] and acceptable [73] in young children. Images will be transmitted to pediatric dentists at the Kodiak who will determine the presence of visible dental caries and (if any) their severity (Table 3).

**Table 3:** Common Risk/Health Factor Measures

Construct	Variable Measure	Source
Bottle/Sippy:	Any (Y/N)	24 h recall (MSB)
	Frequency (#/day)	24 h recall (MSB)
	Added sweet/solid (Y/N)	24 h recall (MSB)
	@ Bed or Nap (Y/N)	24 h recall (MSB)
Dietary Pattern	F & V servings (#/day)	24 h recall (MSB)
	Juice & sweet drinks (#/day)	24 h recall (MSB)
	Sweet & salty snack (#/day)	24 h recall (MSB)
Unique Obesity	Physical activity	Questionnaire
	Screen time	Questionnaire
	BMI-for-Age Z score	Pediatrics visit
	Weight Velocity Z score	Pediatrics visit
Unique Oral Health	Oral hygiene (#/day or wk)	Questionnaire
	Dental visit (Y/N)	Questionnaire
	Visible caries (Y/N)	Intra-oral camera
	Caries Severity (dfs index)	I.O. camera

#### Implementation/Fidelity

Home visiting interventions involve three components: Dose (timing/length of visits); Content (degree to which it was delivered); and Rapport (quality of visitor-participant relationship) [74]. To assess Dose and Content**,** we will adapt a home visit form used in large home visiting studies, for CHALO [75] (see Additional file 1: Figure S1) to be completed after each visit. To assess relationship warmth and rapport, both CHWs and mothers will complete the Working Alliance Questionnaire [76] adapted for the study.

#### Recruitment and retention

Practices will generate quarterly lists of 2–5 month olds seen for well-child visits. Parents will receive an “opt-out” letter from the pediatrician when their child is 4–5 months old, with a number to call to decline study contact. Parents who do not decline will be contacted by study staff to arrange a Baseline (T0) interview. We will set individual practice target recruitment rates based on target population size at each practice. Strategies to promote retention include: gift card incentives for research interviews, quarterly post-card reminders and incentives to notify study of address or name changes, holiday greeting postcards, and reminders for upcoming interviews.

#### Randomization and masking

Participants will be randomized 1:1 to the intervention or EUC control group, after providing informed consent (see below). Following delivery of the EUC at the T0 (baseline) assessment, the CHW will randomize participants by opening a sealed envelope containing the random assignment (prepared in advance by the project statistician). This sequence preserves masking of outcomes assessment at the T0 interview. Neither the outcomes section of the study database, nor written study materials will include a group identifier, though participants may indicate group assignment to research assistants at the12-month (T1) and 18-month (T2) follow-ups.

#### Human subjects

This study was approved by the Albert Einstein college of Medicine’s Committee on Clinical Investigation. Bi-lingual research assistants will consent mothers in their home prior to the baseline (T0) interview.

#### Sample size

Based on preliminary studies, we estimate a between group difference in decline in the number of daily bottles and sippy cups (combined) of 0.7. This equates to a small-moderate standardized effect size (Cohen’s d) of 0.7/2 = 0.35. With *n* = 300 participants (150 per am) the trial will have greater than 85% power to detect an effect size of 0.35 (two-tailed alpha = 0.05)**.** To account for a 20% attrition rate, we will enroll 360 subjects (180 per arm).

Analysis plan: Analyses will be intent-to-treat. Generalized linear mixed models (GLMM) will be employed to analyze primary CRFA outcomes, as well as unique obesogenic and cariogenic outcomes. To test the impact of the intervention on secondary outcomes-- caries, weight for length, velocity of weight gain-- similar GLMM models will be fit. Individuals lost to follow-up at T1 or T2 will remain in the analytic sample. Further details are provided below.

The trial’s primary outcome is frequency of combined sippy cup and bottle use (count/day). The intervention’s effectiveness will be tested by a random-effects Poisson regression model, with the count of bottle/sippy cup as dependent variable, an indicator for study arm, a time indicator and their interaction term as fixed effects, and a random intercept at the person level. We will adjust for child age, given shifts in feeding behavior at this age. If the mean (sd) of the outcome demonstrates over -dispersion relative to a Poisson model, we will use negative binomial regression instead. We will report the coefficient of the interaction term, which estimates the rate ratio in bottle/sippy cup use attributable to the intervention, and its 95% confidence interval. To test the null hypothesis, we will use a z-test.

To test the impact of the intervention on secondary outcomes (behavioral), we will use random-effect generalized linear regression models. The intervention’s impact will be assessed by the coefficient of the interaction term. The estimate and its 95% confidence interval will be reported. For secondary caries, weight for length, and velocity of weight gain outcomes, logistic regression or a linear regression model will be applied as appropriate. *Anthropometrics*: We will use WHO growth standards [77] to calculate *BMI-for-age Z scores* --weight/length^2^ (kg/m^2^), standardized for sex and actual age at measurement. We will categorize children as “overweight” and “obese” if their BMI-for-age Z-scores exceed + 2 and + 3, respectively, as recommended by the WHO [78], and *Weight velocity Z scores*: for each 6 month period: 6 m. > 12 m. 12 m. > 18 m.

**Missing data** will arise from both non-response and study attrition. Random bias created by missing items will be addressed by using multiple imputations [79]. These will use chained equations incorporating baseline and end-of-study data and behavioral variables. For non-random bias, sensitivity analyses will be used to impute best and worst-case credible values for missing variables and then apply the original analyses to these supplemented data sets.

#### Data management

We will use REDCap (Research Electronic Data Capture) software for database development, data entry, coding and secure data storage [80]. Research assistants can access REDCap both onsite and in the field, and enter data directly as it is collected. The team will use REDCap features to manage follow-up reminders, and adherence to T1 and T2 windows for interview completion. All data will be maintained in secure files during and after the trial.

## The CHALO project phase II: Knowledge translation campaign

### Overview

The Knowledge Translation campaign will address SA child health disparities in our region at the community, institutional and policy level.

### Family/community outreach project

We will work with **Sapna NYC**, a nonprofit organization in the Bronx that develops health interventions for SA immigrant families, to develop an *Action Group* community education project. Sapna has used this model in previous programs aimed at breast health screening, nutrition and weight loss [80], and depression [81]. We will develop two Action Groups- one in NJ and one in NY. CHWs will recommend 12 mothers with leadership potential from the intervention to receive an 8-session training that reviews child health and oral health risks, as well as training in leadership, communication, and outreach. CHWS will lead the groups.

### Community outreach

After training, the Action Groups will develop a dissemination project. Elements include: 1) one-on-one outreach (in doctors’ offices, religious institutions, and other community organizations TBD). 2) Community conference/meetings at public libraries or other public spaces. In keeping with past projects, the Action Group will prepare outreach videos for community education. Particularly effective are videos that ‘tell a story’ expressing complex aspects of health behavior and/or illustrate healthy behaviors (e.g. responsive bottle feeding, tooth brushing). Another strategy frequently used in Sapna’s community education projects is the Punthi Path**—**a traditional narrative poem that ‘tells the news’ or relates key information to communities. Action Group members will prepare a Punthi Path on child feeding for use at outreach events, and in the online video.

Outreach will be enhanced with a text messaging alert system which will allow subscribers to receive updates about online content, community events, and other resources. All materials will be available in multiple languages on an informational website developed to be culturally tailored for community members, parents, families and caregivers.

### Professional outreach project

This will include a campaign targeting both clinical providers in pediatrics and oral health, as well as regional and national policy makers. Our partner for this project is the South Asian Total Health Imitative (SATHI), a research and policy institute at Rutgers University. Components include:

1. Kick off Conference. At the beginning of Year 5, SATHI will hold a half-day Global Crossroads Conference focusing on SA child health, nutrition, and oral caries. The conference will be designed to attract physicians, dentists, and other care professionals. A lecture on SA immigrant health, findings from our baseline interviews, a presentation of the community health worker model and implementation experiences in our RCT will be presented, along with emerging results from the RCT. A mother/community member will present on her experiences and issues with feeding. Workshops focusing on practical strategies—best practices, handouts, oral health referrals, counseling checklists--will follow, with a dinner discussion at the end.
2. One on one outreach. Two pediatric residents (Montefiore Residency Program in Social Medicine/Rutgers Dept Pediatrics) will develop one-on-one outreach interventions as part of their required residency projects. They will make on-on-one practice visits to physicians serving SA children in NJ and NY. Checklists, guidelines, patient education materials, and handouts will be developed/adapted and provided to physicians and other practice staff.
3. Traditional Media presentations. We will disseminate information to pediatricians and dentists through write-ups in local professional publications and local SA media (EBC radio, TV Asia etc.).
4. Online education and social media. Late in Year 4, we will develop a website and Facebook page for the project. Educational materials and other relevant materials (guidelines, best practice messages, videos, etc.) will be available on the portal, which will also serve as a core promotional hub for conferences, seminars and other activities. To facilitate knowledge transfer and translation of the CHALO model to the public health community beyond the NY Metropolitan Region, we will present detailed intervention protocol including staff training details, and other findings as they emerge. RCT results will be posted in Year 5.

### Evaluation

We will track attendance, impact (as assessed through knowledge/attitude questionnaires), and participant experiences. Website traffic will be monitored via Google analytics, which will provide metrics on number of site visitors (new and unique), time spent on the website, pages visited, and as well as user demographic information, e.g. gender, age range, geographic location. A pop-up survey of website users may be used to capture additional information, and material downloads will be tracked for reporting. Facebook and YouTube metrics will be captured by those sites’ analytics tools to report views, likes, and basic user demographics. Text messaging campaigns will be managed via the Mobile Commons platform, which reports on number of subscribers, cell carrier, all incoming and outgoing text messages, and all links to content clicked on by subscribers. See Table 4: Evaluation of Knowledge Translation.

**Table 4:** Evaluation of Knowledge Translation

Data Type	Source	Collection/Management
Attendance & demographics	Website & event registration	Event registration; online surveys; Google Analytics; managed in MS Access
Online training Module	Training library	Content, length, delivery method; managed in MS Access
Training/event satisfaction	Evaluation forms	Survey completion at relevant modules; managed in MS Access
Online marketing/Promotion	Mailing/texting/social media lists; web analytics	Mail Chimp – email listserv management and analytics; Mobile Commons – text messaging campaign analytics; Google Analytics – website visit and use.
Knowledge and attitudes	Pre-Posttest surveys	Paper questionnaires delivered as feasible at events and 1-on-1 interactions. Online pre-test via Survey Monkey and/or LMS; managed in MS Access

## Discussion

The Common Risk/Health Factor Approach (CR/HFA) is widely promoted by oral health researchers, policy makers, and the WHO [82–84]. Despite its obvious utility, the CR/HFA has only been applied in a few studies [85–88]. The project described in this paper is the first in the US to use this model as the basis for an intervention. In keeping with the CR/HFA model, CHALO is a multi-level approach. For decades, experts have advocated a multi-level approach to addressing health disparities [89, 90]. Yet to date most interventions, including those for ECC and obesity [91–93], still focus narrowly on individual factors while ignoring interpersonal, institutional and broader levels of influence [94]. CHALO, by contrast, intervenes at each level of the SEM. Many innovations, including the use of intraoral cameras, and culturally adapted dietary measurements, and a public education campaign that includes both traditional and social media, have been incorporated into the design.

CHALO represents the first test of the CR/HFA in a clinical trial in the US. Findings will be submitted for publication in the peer-reviewed literature. An important potential benefit of the CR/HFA approach is cost effectiveness. Future research will examine whether the approach achieves the same or superior results with oral health and obesity outcomes vs.conventional trials focusing on a single outcome, and will compare costs across these approaches to determine cost effectiveness of the CR/HFA.

## Additional file

Additional file 1: **Figure S1.** Example template of recommended content for the schedule of enrolment, interventions, and assessments*. (DOC 49 kb)

## Abbreviations

BMI: Body mass index
CDC: Centers for Disease Control and Prevention
CHW: Community health worker
CR/HFA: Common risk/health factor approach
ECC: Early childhood carries
EUC: Enhanced usual care
MSB: MySmileBuddy
NJ: New Jersey
NY: New York
RCT: Randomized controlled trial
SA: South Asian
UK: United Kingdom
US: United States
WHO: World Health Organization

## References

[1] Satia JA (2010). Dietary acculturation and the nutrition transition: an overview. Appl Physiol Nutr Metab.

[2] Dye BA, Thornton-Evans G (2010). Trends in oral health by poverty status as measured by healthy people 2010 objectives. Public Health Rep.

[3] DenBesten P, Berkowitz R (2003). Early childhood caries: an overview with reference to our experience in California. J Calif Dent Assoc.

[4] Mouradian WE, Wehr E, Crall JJ (2000). Disparities in children's oral health and access to dental care. JAMA : the journal of the American Medical Association.

[5] United States Census B: American Community Survey. In*.*, vol. 2010; 2009.

[6] Asian, Pacific Islanders Health F: South Asians in the United States. In*.*; 2006.

[7] Misra A, Misra R (2003). Asian indians and insulin resistance syndrome: global perspective. Metab Syndr Relat Disord.

[8] Misra A, Singhal N, Sivakumar B, Bhagat N, Jaiswal A, Khurana L (2011). Nutrition transition in India: secular trends in dietary intake and their relationship to diet-related non-communicable diseases. J Diabetes.

[9] Pandit K, Goswami S, Ghosh S, Mukhopadhyay P, Chowdhury S (2012). Metabolic syndrome in south Asians. Indian journal of endocrinology and metabolism.

[10] Misra A, Khurana L (2009). The metabolic syndrome in south Asians: epidemiology, determinants, and prevention. Metab Syndr Relat Disord.

[11] Whincup PH, Gilg JA, Owen CG, Odoki K, Alberti KG, Cook DG (2005). British south Asians aged 13-16 years have higher fasting glucose and insulin levels than Europeans. Diabet Med.

[12] Barnett AH, Dixon AN, Bellary S, Hanif MW, O'Hare JP, Raymond NT, Kumar S (2006). Type 2 diabetes and cardiovascular risk in the UK south Asian community. Diabetologia.

[13] Bansal N, Ayoola OO, Gemmell I, Vyas A, Koudsi A, Oldroyd J, Clayton PE, Cruickshank JK (2008). Effects of early growth on blood pressure of infants of British European and south Asian origin at one year of age: the Manchester children's growth and vascular health study. J Hypertens.

[14] Martinson ML, McLanahan S, Brooks-Gunn J (2015). Variation in child body mass index patterns by race/ethnicity and maternal nativity status in the United States and England. Matern Child Health J.

[15] Bedi R, Uppal RD (1995). The oral health of minority ethnic communities in the United Kingdom. Br Dent J.

[16] Wong F. Epidemiology: inequalities in oral health for deprived multiethnic communities. Br Dent J. 2000;189(2):84–4.

[17] Prendergast MJ, Beal JF, Williams SA (1997). The relationship between deprivation, ethnicity and dental health in 5-year-old children in Leeds, UK. Community Dent Health.

[18] Gray M, Morris AJ, Davies J (2000). The oral health of south Asian five-year-old children in deprived areas of Dudley compared with white children of equal deprivation and fluoridation status. Community Dent Health.

[19] Yin HS, Sanders LM, Rothman RL, Shustak R, Eden SK, Shintani A, Cerra ME, Cruzatte EF, Perrin EM (2014). Parent health literacy and "obesogenic" feeding and physical activity-related infant care behaviors. J Pediatr.

[20] Bonuck K, Kahn R, Schechter C (2004). Is late bottle-weaning associated with overweight in young children? Analysis of NHANES III data. Clin Pediatr.

[21] Gooze RA, Anderson SE, Whitaker RC (2011). Prolonged bottle use and obesity at 5.5 years of age in US children. J Pediatr.

[22] Kimbro RT, Brooks-Gunn J, McLanahan S (2007). Racial and ethnic differentials in overweight and obesity among 3-year-old children. Am J Public Health.

[23] Behrendt A, Sziegoleit F, Muler-Lessmann V, Ipek-Ozdemir G, Wetzel WE (2001). Nursing-bottle syndrome caused by prolonged drinking from vessels with bill-shaped extensions. ASDC J Dent Child.

[24] Kaste LM, Gift HC (1995). Inappropriate infant bottle feeding. Status of the Healthy People 2000 Objective. Arch Pediatr Adolesc Med.

[25] Woo Baidal JA, Locks LM, Cheng ER, Blake-Lamb TL, Perkins ME, Taveras EM (2016). Risk factors for childhood obesity in the first 1,000 days: a systematic review. Am J Prev Med.

[26] Carvalho JC, Silva EF, Vieira EO, Pollaris A, Guillet A, Mestrinho HD (2014). Oral health determinants and caries outcome among non-privileged children. Caries Res.

[27] Almquist-Tangen G, Dahlgren J, Roswall J, Bergman S, Alm B (2013). Milk cereal drink increases BMI risk at 12 and 18 months, but formula does not. Acta paediatrica.

[28] Hyden CBK (2014). Addition of solids and sweeteners in toddler bottles and sippy cups. ICAN: Infant, Child, & Adolescent Nutrition.

[29] Lumeng JC, Taveras EM, Birch L, Yanovski SZ (2015). Prevention of obesity in infancy and early childhood: a National Institutes of Health workshop. JAMA Pediatr.

[30] Li R, Scanlon KS, May A, Rose C, Birch L (2014). Bottle-feeding practices during early infancy and eating behaviors at 6 years of age. Pediatrics.

[31] Dixon B, Pena MM, Taveras EM (2012). Lifecourse approach to racial/ethnic disparities in childhood obesity. Adv Nutr.

[32] Li R, Magadia J, Fein SB, Grummer-Strawn LM (2012). Risk of bottle-feeding for rapid weight gain during the first year of life. Arch Pediatr Adolesc Med.

[33] Faruque AS, Ahmed AM, Ahmed T, Islam MM, Hossain MI, Roy SK, Alam N, Kabir I, Sack DA (2008). Nutrition: basis for healthy children and mothers in Bangladesh. J Health Popul Nutr.

[34] Godson JH, Williams SA (1996). Oral health and health related behaviours among three-year-old children born to first and second generation Pakistani mothers in Bradford, UK. Community Dent Health.

[35] Watt RG (2000). Oral health promotion: a national survey of infant feeding in Asian families: summary of findings relevant to oral health. Br Dent J.

[36] Watt RG (2000). A national survey of infant feeding in Asian families: summary of findings relevant to oral health. Br Dent J.

[37] Williams SA, Hargreaves JA (1990). An inquiry into the effects of health related behaviour on dental health among young Asian children resident in a fluoridated city in Canada. Community Dent Health.

[38] Engle PL, Pelto GH (2011). Responsive feeding: implications for policy and program implementation. J Nutr.

[39] Worobey J, Lopez MI, Hoffman DJ (2009). Maternal behavior and infant weight gain in the first year. J Nutr Educ Behav.

[40] Farrow C, Blissett J (2006). Does maternal control during feeding moderate early infant weight gain?. Pediatrics.

[41] Hurley KM, Cross MB, Hughes SO (2011). A systematic review of responsive feeding and child obesity in high-income countries. J Nutr.

[42] Karasz A, Patel V, Ranasinghe S, Chaudhuri K, McKee MD (2014). Preventing caries in young children of immigrant Bangladeshi families in New York: perspectives of mothers and paediatricians. Community Dent Health.

[43] Khunti K, Stone MA, Bankart J, Sinfield PK, Talbot D, Farooqi A, Davies MJ (2007). Physical activity and sedentary behaviours of south Asian and white European children in inner city secondary schools in the UK. Fam Pract.

[44] Khunti K, Stone MA, Bankart J, Sinfield P, Pancholi A, Walker S, Talbot D, Farooqi A, Davies MJ (2008). Primary prevention of type-2 diabetes and heart disease: action research in secondary schools serving an ethnically diverse UK population. J Public Health (Oxf).

[45] Palaniappan L, Anthony MN, Mahesh C, Elliott M, Killeen A, Giacherio D, Rubenfire M (2002). Cardiovascular risk factors in ethnic minority women aged < or =30 years. Am J Cardiol.

[46] Lawton J, Ahmad N, Hanna L, Douglas M, Hallowell N (2006). 'I can't do any serious exercise': barriers to physical activity amongst people of Pakistani and Indian origin with type 2 diabetes. Health Educ Res.

[47] Williams SA, Ahmed IA, Hussain P (1991). Ethnicity, health and dental care--perspectives among British Asians: 1. Dental update.

[48] Williams SA, Ahmed IA, Hussain P (1991). Ethnicity, health and dental care--perspectives among British Asians: 2. Dental update.

[49] Patel VV, Rajpathak S, Karasz A. Bangladeshi immigrants in New York City: a community based health needs assessment of a hard to reach population. J Immigr Minor Health. 2011;5:1–7. 10.1007/s10903-011-9555-5 PMC4885680 22116745

[50] Hussain F, Cochrane R (2004). Depression in south Asian women living in the UK: a review of the literature with implications for service provision. TranscultPsychiatry.

[51] Karasz A (2005). Cultural differences in conceptual models of depression. Soc Sci Med.

[52] Hurley KM, Black MM, Papas MA, Caulfield LE (2008). Maternal symptoms of stress, depression, and anxiety are related to nonresponsive feeding styles in a statewide sample of WIC participants. J Nutr.

[53] Goulding AN, Rosenblum KL, Miller AL, Peterson KE, Chen YP, Kaciroti N, Lumeng JC (2014). Associations between maternal depressive symptoms and child feeding practices in a cross-sectional study of low-income mothers and their young children. The international journal of behavioral nutrition and physical activity.

[54] Lumeng JC, Castle VP, Lumeng CN (2010). The role of pediatricians in the coordinated national effort to address childhood obesity. Pediatrics.

[55] Henderson EJ. Acceptability of delivery of dietary advice in the dentistry setting to address obesity in pre-school children: a case study of the common risk factor approach. Public Health Nutr. 2014:1–6. 10.1017/S1368980014002249 PMC10271493 25335822

[56] Isong I, Dantas L, Gerard M, Kuhlthau K. Oral health disparities and unmet dental needs among preschool children in Chelsea, MA: exploring mechanisms, defining solutions. Journal of oral hygiene & health. 2014;2 10.4172/2332-0702.1000138 PMC4299657 25614878

[57] Long CM, Quinonez RB, Beil HA, Close K, Myers LP, Vann WF, Rozier RG (2012). Pediatricians' assessments of caries risk and need for a dental evaluation in preschool aged children. BMC Pediatr.

[58] American Academy of Pediatrics: Bright futures: Guidelines for Health Supervision of Infants, Children, and Adolescents, vol 3; 2008.

[59] Dentistry AAoP (2013). Guideline on periodicity of examination, preventive dental services, anticipatory guidance/counseling, and oral treatment for infants, children, and adolescents. Pediatr Dent.

[60] Section On oral H (2014). Maintaining and improving the oral health of young children. Pediatrics.

[61] Institute of M, National Research C (2011). Improving access to oral health Care for Vulnerable and Underserved Populations.

[62] Hakim RB, Babish JD, Davis AC (2012). State of dental care among Medicaid-enrolled children in the United States. Pediatrics.

[63] Peacock S, Konrad S, Watson E, Nickel D, Muhajarine N (2013). Effectiveness of home visiting programs on child outcomes: a systematic review. BMC Public Health.

[64] Bryans A, Cornish F, McIntosh J (2009). The potential of ecological theory for building an integrated framework to develop the public health contribution of health visiting. Health Soc Care Community.

[65] Peterson KE, Sorensen G, Pearson M, Hebert JR, Gottlieb BR, McCormick MC (2002). Design of an intervention addressing multiple levels of influence on dietary and activity patterns of low-income, postpartum women. Health Educ Res.

[66] Levine J, Wolf RL, Chinn C, Edelstein BL (2012). MySmileBuddy: an iPad-based interactive program to assess dietary risk for early childhood caries. J Acad Nutr Diet.

[67] Custodio-Lumsden CL, Wolf RL, Contento IR, Basch CE, Zybert PA, Koch PA, Edelstein BL (2016). Validation of an early childhood caries risk assessment tool in a low-income Hispanic population. J Public Health Dent.

[68] Splieth CH, Nourallah AW (2006). An occlusal plaque index. Measurements of repeatability, reproducibility, and sensitivity. Am J Dent.

[69] Kopycka-Kedzierawski DT, Billings RJ, McConnochie KM (2007). Dental screening of preschool children using teledentistry: a feasibility study. Pediatr Dent.

[70] Kopycka-Kedzierawski DT, Billings RJ (2006). Teledentistry in inner-city child-care centres. J Telemed Telecare.

[71] Kopycka-Kedzierawski DT, Bell CH, Billings RJ (2008). Prevalence of dental caries in early head start children as diagnosed using teledentistry. Pediatr Dent.

[72] Elfrink ME, Veerkamp JS, Aartman IH, Moll HA, Ten Cate JM (2009). Validity of scoring caries and primary molar hypomineralization (DMH) on intraoral photographs. Eur Arch Paediatr Dent.

[73] Boye U, Foster GR, Pretty IA, Tickle M (2012). Children's views on the experience of a visual examination and intra-oral photographs to detect dental caries in epidemiological studies. Community Dent Health.

[74] Paulsell D, Boller K, Hallgren K, Esposito A (2010). Assessing home visit quality: Dosage,content,and relationships. Zero to Three*.* Mathematical policy research.

[75] Barrett K, Zaveri H, Strong D: Fidelity data collection manual for the evidence-based home visiting to prevent child maltreatment cross-site evaluation. In*.* Edited by Children's Bureau AfCaF, US Department of Health and Human Services. Princeton, NJ: Mathematica Policy Research; 2010.

[76] Santos R (2005). Development and validation of a revised short version of the working alliance inventory.

[77] Group WHOMGRS (2006). WHO child growth standards based on length/height, weight and age. Acta Paediatr Suppl.

[78] Organization WH (2008). WHO child growth standards: training course on child growth assessment.

[79] White IR, Royston P, Wood AM (2011). Multiple imputation using chained equations: issues and guidance for practice. Stat Med.

[80] Karasz A, Patel VV, Chaudhury K, Kabita M, Jaiman A (2013). The APPLE obesity project: using Community of Practice Theory to build a sustainable, community based Interventon (submitted).

[81] Karasz A, Raghavan S, Ranasinghe K, Kabita: The ASHA project: an innovative depression treatment intervention based on asset theory. In*.*

[82] Watt RG, Sheiham A (2012). Integrating the common risk factor approach into a social determinants framework. Community Dent Oral Epidemiol.

[83] Sheiham A, Watt RG (2000). The common risk factor approach: a rational basis for promoting oral health. Community Dent Oral Epidemiol.

[84] Moynihan P, Makino Y, Petersen PE, Ogawa H. Implications of WHO guideline on sugars for dental health professionals. Community Dent Oral Epidemiol. 2017;46(1):1-7. 10.1111/cdoe.12353 29168887

[85] Do LG, Scott JA, Thomson WM, Stamm JW, Rugg-Gunn AJ, Levy SM, Wong C, Devenish G, Ha DH, Spencer AJ (2014). Common risk factor approach to address socioeconomic inequality in the oral health of preschool children--a prospective cohort study. BMC Public Health.

[86] Arrow P, Raheb J, Miller M (2013). Brief oral health promotion intervention among parents of young children to reduce early childhood dental decay. BMC Public Health.

[87] Maupome G, Karanja N, Ritenbaugh C, Lutz T, Aickin M, Becker T (2010). Dental caries in American Indian toddlers after a community-based beverage intervention. Ethn Dis.

[88] Karanja N, Lutz T, Ritenbaugh C, Maupome G, Jones J, Becker T, Aickin M (2010). The TOTS community intervention to prevent overweight in American Indian toddlers beginning at birth: a feasibility and efficacy study. J Community Health.

[89] Green LW, Richard L, Potvin L (1996). Ecological foundations of health promotion. American journal of health promotion : AJHP.

[90] Stokols D (2000). Social ecology and behavioral medicine: implications for training, practice, and policy. Behav Med.

[91] Pratt CA, Boyington J, Esposito L, Pemberton VL, Bonds D, Kelley M, Yang S, Murray D, Stevens J (2013). Childhood obesity prevention and treatment research (COPTR): interventions addressing multiple influences in childhood and adolescent obesity. Contemp Clin Trials.

[92] Penn S, Kerr J (2014). Childhood obesity: the challenges for nurses. Nurs Child Young People.

[93] Baur LA, O'Connor J (2004). Special considerations in childhood and adolescent obesity. Clin Dermatol.

[94] Golden SD, Earp JA (2012). Social ecological approaches to individuals and their contexts: twenty years of health education & behavior health promotion interventions. Health Educ Behav.

