# Supplementary material for: Reducing pediatric caries and obesity risk in South Asian immigrants: randomized controlled trial of common health/risk factor approach
Source: BMC Public Health. 2018 May 31;18:680. doi: 10.1186/s12889-018-5317-9 (PMC5984363; doi:10.1186/s12889-018-5317-9)
Supplement: Supplementary file 1 — Figure S1. Example template of recommended content for the schedule of enrolment, interventions, and assessments*. (DOC 49 kb) [file 12889_2018_5317_MOESM1_ESM.doc]

Figure S1 Example template of recommended content for the schedule of enrolment, interventions, and assessments.*

|  | **STUDY PERIOD** | | | | | | | |
| --- | --- | --- | --- | --- | --- | --- | --- | --- |
|  | **Enrolment** | **Allocation** | **Post-allocation** | | | | | **Close-out** |
| **TIMEPOINT**** | ***-t_1_*** | **0** | ***t_1_*** | ***t_2_*** | ***t_3_*** | ***t_4_*** | ***etc.*** | ***t_x_*** |
| **ENROLMENT:** |  |  |  |  |  |  |  |  |
| **Eligibility screen** | X |  |  |  |  |  |  |  |
| **Informed consent** | X |  |  |  |  |  |  |  |
| ***[List other procedures]*** | X |  |  |  |  |  |  |  |
| **Allocation** |  | X |  |  |  |  |  |  |
| **INTERVENTIONS:** |  |  |  |  |  |  |  |  |
| ***[Intervention A]*** |  |  |  |  |  |  |  |  |
| ***[Intervention B]*** |  |  | X |  | X |  |  |  |
| ***[List other study groups]*** |  |  |  |  |  |  |  |  |
| **ASSESSMENTS:** |  |  |  |  |  |  |  |  |
| ***[List baseline variables]*** | X | X |  |  |  |  |  |  |
| ***[List outcome variables]*** |  |  |  | X |  | X | etc. | X |
| ***[List other data variables]*** |  |  | X | X | X | X | etc. | X |

*Recommended content can be displayed using various schematic formats. See SPIRIT 2013 Explanation and Elaboration for examples from protocols.

**List specific timepoints in this row.
